# The colonic interleukin-19 aggravates the dextran sodium sulfate/stress-induced comorbidities due to colitis and anxiety

**DOI:** 10.3389/fimmu.2023.1153344

**Published:** 2023-03-02

**Authors:** Qiongyu Li, Fantao Meng, Xiangxian Ma, Zhe Sun, Juanjuan Dai, Jing Liu, Dan Li, Peijia Cong, Ruixue Xu, Di Zhao, Wentao Wang, Dan Wang, Cuilan Liu, Faxiang Wang, Chen Li, Haifeng Lian

**Affiliations:** ^1^ Department of Gastroenterology, Binzhou Medical University Hospital, Binzhou, China; ^2^ Medical Research Center, Binzhou Medical University Hospital, Binzhou, China; ^3^ Institute for Metabolic & Neuropsychiatric Disorders, Binzhou Medical University Hospital, Binzhou, China; ^4^ Department of Psychology, Binzhou Medical University Hospital, Binzhou, China; ^5^ College of Nursing, Binzhou Medical University, Binzhou, China; ^6^ Department of Neurology, Xinqiao Hospital, Third Military Medical University, Chongqing, China

**Keywords:** inflammatory bowel disease, anxiety, colitis, interleukin-19, hippocampus, brain-derived neurotrophic factor

## Abstract

Comorbidities due to inflammatory bowel disease (IBD) and anxiety are commonly acknowledged; however, their underlying basis is unclear. In the current study, we first conducted a clinical retrospective analysis to identify the enhancive incidence rate of IBD before or after the epidemic of Corona Virus Disease 2019 (COVID-19), with higher Generalized Anxiety Disorder-7 (GAD-7), as well as poorer Gastrointestinal Quality of Life Index (GIQLI). Then, the dextran sodium sulfate (DSS) and chronic unpredictable stress (CUS)-induced IBD and anxiety comorbid models were established with the correlational relations between symptoms of IBD and anxiety-related behaviors. We found dysfunctional up-regulation of a new inflammatory factor interleukin (IL)-19 in the colon of DSS/CUS treated mice. Overexpression of IL-19 in colon induced anxious phenotypes, and accelerated the anxious condition and symptoms of colitis in the DSS/CUS model by promoting the expression of inducible nitric oxide synthase (iNOS), IL-1β, and IL-6 pro-inflammatory factors, and activating signal transducer and activator of transcription 3 (STAT3) signaling pathway in the colon. Furthermore, overexpression of IL-19 in the colon also reduced the expression levels of brain-derived neurotrophic factor (BDNF), extracellular signal-regulated kinase (ERK), and cAMP-response element binding protein (CREB) signaling pathways activity in the hippocampus. These results suggest that IL-19 was a pivotal player in DSS/CUS-induced comorbidities of colitis and anxiety with different signaling pathways for the colon and hippocampus, which provides a candidate gene to explore the pathophysiology of comorbidities due to colitis and anxiety.

## Introduction

Inflammatory bowel disease (IBD) is an idiopathic, chronic and recurrent intestinal disease with complicated pathogenesis, mainly characterized by ulcerative colitis (UC) and crohn’s disease (1, 2). Its etiology is currently believed to be associated with a complex interaction between genetic and environmental variables (2, 3). The existence of influential reciprocal processes between IBD and morbidity due to psychiatric diseases, such as anxiety or depression, is commonly acknowledged (4, 5). Since individuals with IBD are more likely to suffer from anxiety or/and depression, these may be sufficient to trigger or exacerbate health conditions in IBD (6, 7). Nevertheless, how these conditions affect each other remains uncertain.

The brain-gut axis is a term used to explain the underlying interactions between the central system and the gastrointestinal tract; in brief, psychological disorders influence gastrointestinal function *via* stress reactivity, which results in the activation of the hypothalamus-pituitary-adrenal axis to regulate the gastrointestinal tract, such as increased intestinal permeability by the release of adrenocorticotropic hormone (5, 8). The increased intestinal permeability might also allow the gut microbiota or other secreting factors to interact with the nervous system by the vagus nerve or external secretion system, such as inflammatory factors (8). However, the specific mechanism between them has not been fully clarified.

Interleukin-19 (IL-19), a member of the IL-10 cytokine family, has sequence homology with IL-10 (9, 10). They all send signals through the heterodimer IL-20R to activate the signal transducer and STAT3 signal pathway (11). IL-20Rb can be paired with IL-20Ra to form a type I IL-20R to allow IL-19 signal transduction (12). It was reported that IL-19 might play an essential role in inflammation through the up-regulation of IL-6 and inducing apoptosis (13). Certain literature has shown that endotoxins and stress-induced anxiety can enhance the expression of IL-19, while β-adrenergic receptor antagonists can block the stress-induced increase in IL-19 expression (14). The expression of IL-19 in patients with active IBD was significantly increased, and the expression of IL-19 in the peripheral blood of mice with colitis induced by dextran sodium sulfate (DSS) was also increased (15); however, the concrete role of IL-19 leading to comorbidities due to colitis and anxiety is still undetermined, and requires further exploration.

In the present study, we first identified the morbidities related to IBD and the prevalence of anxiety before or after the outbreak of the COVID-19 pandemic *via* a retrospective analysis. A DSS/CUS comorbid model of colitis and anxiety was established to demonstrate whether IL-19 was associated with these comorbidities. Then, we measured the anxiety-related behaviors and the symptoms of IBD after overexpression of IL-19 in colon; furthermore, we evaluated how IL-19 affects the sensitivity of the DSS/CUS-induced comorbid anxious behaviors; the pathological severities and their molecular pathways were determined.

## Materials and methods

### Retrospective analysis

We performed a retrospective analysis using the Inpatient Medical Record Database of Binzhou Medical University Hospital. Medical records of three-month periods, i.e., March 1, 2019, to May 31, 2019 (27 patients), November 1, 2019, to January 31, 2020 (19 patients) before the COVID-19 pandemic, and March 1, 2020, to May 31, 2020 (during COVID-19 pandemic) (42 patients), November 1, 2020, to January 31, 2021 (31 patients) during COVID-19 pandemic were collected. Two prior times were selected for comparison to minimize any seasonal differences. Within the inpatient population in the Gastroenterology Department, we identified all patients with a diagnosis of IBD (UC) based on the eighth and tenth revisions of the International Classification of Diseases, and calculated the incidence rates based on total inpatients. In addition, we searched the Outpatient Gastrointestinal Endoscopy Database of Binzhou Medical University Hospital from the four aforementioned periods for diagnosis of IBD (UC) with colonoscopy. We compared the detection rates of IBD in all outpatients who underwent colonoscopy between these periods.

### Participant recruitment

This study was carried out with the approval of the local Ethics Committee of Binzhou Medical University Hospital, and all participants signed written informed consent. We recruited 32 IBD patients and 44 healthy controls. All patients were treated at the Gastroenterology Department of Binzhou Medical University Hospital from March 2020 to May 2020. IBD was diagnosed by gastroenterologists specialized in treating patients with IBD. The control volunteers were recruited openly or from asymptomatic colonoscopies that underwent a physical examination. They all have regular follow-up. Exclusion criteria included a history of malignant tumors, communication disorders such as verbal expression, and severe psychiatric disorders.

### Questionnaires

All participants were allowed to complete the following two questionnaires: 1). Generalized Anxiety Disorder-7 (GAD-7): GAD-7 is a self-rating scale for anxiety screening of participants through seven questions over the past two weeks, each with four options of “no”, “several days”, “more than half of the time” and “almost every day”, corresponding to 0, 1, 2 and 3 respectively. In the total score, 0-4 points as no anxiety, 5-9 points as mild anxiety, 10-14 points as moderate anxiety, ≥ 15 points as severe anxiety. 2). Gastrointestinal Quality of Life Index (GIQLI): The quality-of-life scale assesses the patient’s actual quality-of-life appearance. The questionnaire has 36 items, based on a five-choice scale (four = not at all, three = slightly, two = moderately, one = quite a bit, zero = extremely), and the higher the score, the better the quality of life.

### Animals

Eight-week-old male C57BL/6 mice (20-25g) were purchased from Jinan Pengyue Experimental Animal Breeding Co Ltd (China; License No. SYXK [Lu] 20190003). All mice were housed in cages of up to five mice with free access to food and water at 22 ± 2°C and 55 ± 5% relative humidity, and the animal experiments were approved by the Animal Ethics Committee of the Binzhou Medical University Hospital, and the experimental scheme is carried out strictly in accordance with the Animal (Scientific procedure) Act of 1986 and related guidelines (16).

### Drug administration

DSS (MP Biomedicals, Solon, USA) was orally administered in drinking water (2.5% w/v) to induce colonic inflammation.

### Disease activity index

DAI is an index used to assess the severity of colitis. Mice needed to be weighed daily; stool properties and rectal bleeding were observed to assess DAI, which was scored 0-4 for each of these three items as detailed in **Supplementary Table 1** (17, 18). Among them, fecal occult blood was detected using a fecal occult blood test kit (Guangzhou Sijia Biotechnology Co., Ltd., China), and DAI was the sum of the percentage of weight loss, stool consistency and fecal bleeding.

### Overexpression of IL-19, colonic perfusion, and fluorescence analysis

The coding region of mouse IL-19 (NM_001009940) with cytomegalovirus enhancer fused to cytomegalovirus promoter was packaged into Adeno-associated virus (AAV) (AAV-IL-19-GFP) with titer > 1 × 10^12^ vg/mL (Hanbio, Shanghai, China), and AAV-GFP was used as the non-specific control. After fasting overnight, the catheter was smeared with paraffin oil. After anesthesia, it was slowly inserted into the colon of mice, and 200 μL AAV (6 × 10^11^ vg/mL) was slowly injected into the colon. Behavioral tests were performed after 21 days. Then, these mice were transcardiac perfused with ice-cold phosphate-buffered saline and 4% paraformaldehyde, the colon were post-fixed in 4% paraformaldehyde and dehydrated in 30% sucrose. Frozen colon tissue sections were prepared, which were mounted on polylysine-coated glass slides and cover-slipped using fluorescence mounting medium. Finally, the images were captured using an Olympus FV1000 confocal microscope (Olympus, Shinjuku, Tokyo, Japan) (19).

### Chronic unpredictable stress

The CUS procedure was carried out as described (20, 21), with minor modifications. Briefly, this stress model aims to expand the unpredictability and moderate stress intensity. This experimental strategy consisted of various stressors (including restraint stress for 2 h, tail pinch for 15 min, constant light for 24 h, a high platform for 30 min, wet bedding for 24 h, and electric shock for 10 min), and two stressors were applied randomly at different times of the day.

### Behavioral tests

#### Sucrose preference test

The SPT procedure was conducted as described (20). Before the test, two bottles of water were placed in a home cage for a week. During the test, each mouse in each cage was placed with pure water on one side and 1% w/v sucrose solution on the opposite side. The sucrose preference is expressed as a percentage of sucrose solution with the total liquid consumption.

#### Elevated plus maze test

EPM test is used to detect the degree of anxiety based upon the contradiction between the high fear of the mice and the exploration characteristics of the new environment, according to the previous report (22). The elevated cross maze is a cross-shaped device consisting of four arms (30 cm long and 5 cm wide). The device is placed about 70 cm above the ground, with walls 12 cm high on both sides and ends of the two closed arms. The sides and ends of the two open arms are open without walls, and there is a 5× 5 cm^2^ middle area at the intersection of the four arms. During the test, the mice were placed in the middle area with their head facing the junction of the open arm and the closed arm, to allow the mice to explore the open arm or the closed arm freely; the activity of the mice was recorded for five minutes with a camera mounted above the maze. The time spent in the open arm (TSOA), the number of entries into the open arm (NEOA), and the numbers of entries into each arm (NEEA) were recorded manually. The percentage of these three indicators is used to assess the degree of anxiety.

#### Light–dark box test

The LD test is conducted to elevate the degree of anxiety with the contradiction of natural fear of the bright environment and exploration of the novel environment (22). The experimental device includes an open box with a size of 27 × 27 × 30 cm^3^ and a dark box with a size of 18 × 27 × 30 cm^3^. A resin partition with a small hole separates the open and dark boxes. Mice can travel freely between open boxes and dark boxes by the small hole. The light of the bright box is 700lux, and the dark box is kept dark and covered with a black opaque board. Mice were placed individually in the center of the dark compartment facing away from the door, and the mouse was considered to enter when all four claws were in illuminated compartment. the latency of enter illuminated compartment (LIC), the time spent in illuminated compartment (TSIC), and the counts between illuminated and dark compartments (CIDC) were collected for five minutes to evaluate the anxiety state of mice with a video camera placed above the light box.

#### Open field test

OFT is a model to evaluate animal anxiety behavior based on the contradiction between the desire to explore a new environment and their fear of open conditions (23). The box height of the open field is 40 cm, the size of the site is 60 × 60 cm^2^, and the central area is defined as 20 × 20 cm^2^, which is always illuminated. The mice were initially placed in the middle of the open field, and the movement area and trajectory of the mice for five minutes were observed through a camera. Clean the instrument with 20% alcohol before each test. The data were analyzed by Any-maze software (Stoelting, Wood Dale, IL, USA). Record the activity of mice with time spent in central zone (TSCZ), total distance in central zone (TDCZ), and total distance traveled (TDT).

#### Locomotor activity

Motor activity is measured in a SuperFlex Fusion open-air cage (40 × 40 × 30 cm^3^, Omnitech Electronics Inc., Columbus, OH, USA). The animals were gently placed in the center of the test apparatus and allowed to explore the field for 30 min. The movement of the mice was monitored using an infrared light sensor mounted on the cage, and the total distance traveled was analyzed using Fusion software (Omnitech Electronics Inc., Columbus, OH, USA) (22).

### Visceral sensitivity

The mice fasted for one day before the test, but were allowed to drink freely. After anesthesia, the balloon was gently inserted into the colon. A colorectal dilatation test was performed on the abdominal withdrawal reflex until the mice were awake and adapted to the environment. The balloon pressure quickly reached 20, 40, 60, and 80 mmHg. When each pressure gradient was maintained for 10 sec, the abdominal withdrawal reflex score was; 0: emotionally stable; 1: restlessness and occasionally twisted their heads; 2: slight contraction of the abdominal and dorsal muscles, but the abdomen was not lifted from the ground; 3: abdominal back muscles strongly contracted, and the abdomen was lifted off the ground; 4: the mice’s abdomen was arched and lifted the abdomen and perineum off the ground. The pressure value was taken as the visceral pain threshold, and each mouse was measured three times at 10-minute intervals, taking the abdominal lift off the ground as the standard (24).

### Quantitative real-time PCR analysis

Total ribonucleic acid (RNA) was extracted using the Tissue RNA kit (Omega, Doraville, GA, USA). Total RNA was reverse transcribed into complementary DNAs (cDNAs) with HiScript II QRT SuperMix (Vazyme, Nanjing, China), which was applied for real-time PCR test by the StepOnePlus real-time PCR system under the conditions as previously descripted (23) (Applied Biosystems, Waltham, MA, USA). Primer sequences used to amplify each gene are listed in **Supplemental Table 2**. The housekeeping gene β-tubulin or β-actin was used as a reference gene for normalizing gene expression. The results were analyzed by 2−ΔΔCT method (25).

### Western blot

The Western blot protocol was conducted as previously descripted (20) In briefly, total protein from the tissues was extracted using radioimmunoprecipitation assay lysis buffer (Beyotime, Nanjing, China) containing the phosphatase inhibitor PhosStop (Roche, Germany) and protease inhibitor phenylmethyl sulfonyl fluoride (Beyotime, Nanjing, China). Lysates were homogenized using a tissue homogenizer. After centrifugation, the protein concentration of the supernatants was determined using a bicinchoninic acid protein assay kit. The protein samples were separated using sodium dodecyl sulfate-polyacrylamide gel, and transferred to a polyvinylidene difluoride membrane. After blocking with 5% nonfat dry milks, the primary antibodies anti-brain-derived neurotrophic factor (BDNF) (ab108319, 1:1000, Abcam, Cambridge, UK), anti-ERK1/2 (#9102, 1:1000, Cell Signaling Technology, Danvers, MA, USA), anti-p-ERK1/2 (Thr202/Tyr204; #4370, 1:1000, Cell Signaling Technology), anti-β-actin (#4970, 1:1000, Cell Signaling Technology), anti-cAMP-response element binding protein (CREB) (#9197, 1:1000, Cell Signaling Technology), anti-p-CREB (Ser133; #9198, 1:1000, Cell Signaling Technology), anti-STAT3 (#9139, 1:1000, Cell Signaling Technology), and anti-p- STAT3 (#9145, 1:1000, Cell Signaling Technology) were added and allowed to incubate at 4°C overnight. The membranes were washed with Tris-Buffered Saline and incubated with goat anti-rabbit IRDye 680LT (#926-68021, 1:5,000, Li-COR Biosciences, Lincoln, NE, USA) or goat anti-mouse IRDye 800CW (#926-32210, 1:5,000, Li-COR Biosciences) fluorescent secondary antibodies for 90 min at room temperature and visualized using the Odyssey Infrared Imaging System (Li-COR Biosciences).

### Statistical analysis

Statistical software GraphPad Prism 8 was used to perform all statistical analyses. The Shapiro-Wilk and F-test were used for normality and equal variance assumptions, respectively. Two-tailed unpaired t-tests were applied for normally distributed data. Two-tailed unpaired t-tests with Welch’s correction were used for normally distributed data with unequal variances. Mann-Whitney U tests were used for the non-normally distributed data. One-way analysis of variance and Sidak *post hoc* tests were used to analyze three or more groups. The linearity between the two variables was assessed by calculating Pearson’s correlation coefficient. The counting data were expressed in the number of cases (n) and percentage (%), and the chi-square test compared the counting data. A value of p<0.05 indicates statistical significance. The behavioral results are derived from two independent experiments. All data are expressed as mean ± standard error of the mean.

## Results

### The COVID-19 pandemic significantly raised the incidence rate of IBD

To investigate the causal relation between external stress and the incidence of IBD, we explored the Inpatient Medical Record Database of Binzhou Medical University Hospital by addressing the targeted periods before or during the COVID-19 pandemic. We observed that before COVID-19, there were 27 IBD patients out of 2065 inpatients from March 1 to May 31, 2019; November 1, 2019, to January 31, 2020, 19 out of 1944 inpatients suffered IBD (total incidence rate: 1. 1474%). During the COVID-19 pandemic, there were 42 patients with UC out of 1792 inpatients with an incidence rate was 2.3437% from March 1 to May 31, 2020, there were 31 patients with UC out of 1834 inpatients with an incidence rate was 1.6903% from November 1, 2020, to January 31, 2021(total incidence rate: 2.0132%), which was higher than that before COVID-19 (incidence rate: 1. 1474%) (p=0.0023, χ^2^ = 9.3020, odds ratio (OR): 0.5649, 95% confidence interval (CI): 0.3858 to 0.8149). There were no significant difference regarding age and gender (gender: p=0.8676, χ^2^ = 0.0596, OR: 0.9120, 95% CI: 0.4420 to 1.8550) (**Table 1**).

**Table 1:** Percentages of IBD patients in all inpatients and outpatients before and during the COVID-19.

	Before COVID-19	During COVID-19	P	χ2	OR	95% CI
**Inpatients** **n (%)**	46 (1.1474)	73 (2.0132)	0.0023	9.3020	0.5649	0.3858 to 0.8149
**Age** **mean ± SEM**	50.7200 ± 2.0010	51.7800 ± 1.8150	P=0.7032			
**Female**	21 (45.6522)	35 (47.9452)	0.8676	0.0596	0.9120	0.4420 to 1.8550
**Male**	25 (54.3478)	38 (52.0548)				
**Outpatient** **n (%)**	23 (1.5797)	46 (3.2928)	0.0029	8.8660	0.4714	0.2797 to 0.7775
**Age** **mean ± SEM**	49.7800 ± 2.9290	48.3500 ± 2.3790	P=0.7178			
**Female**	8 (34.7826)	19 (41.3043)	0.6008	0.2738	0.7579	0.2513 to 2.2350
**Male**	15 (65.2174)	27 (58.6957)				

Furthermore, we compared the incidence rate of IBD in outpatient gastrointestinal endoscopy rooms during these periods. Before COVID-19, there were 16 IBD patients out of 942 outpatients from March 1 to May 31, 2019, and 7 out of 514 outpatients from November 1, 2019, to January 31, 2020, suffered IBD (total incidence rate: 1.5797%). During the COVID-19 pandemic, 24 patients with UC out of 654 outpatients from March 1 to May 31, 2020 with a detection rate of 3.6697% were found, 22 patients with UC out of 743 outpatients from November 1, 2020, to January 31, 2021 with a detection rate of 2.9610% were found (total incidence rate: 3.2928%), which was significantly higher than that before COVID-19 (incidence rate: 1.5797%) (p=0.0029, χ^2^ = 8.8660, OR: 0.4714, 95% CI: 0.2797 to 0.7775). There was no significant difference regarding age and gender (gender: p=0.6008, χ^2^ = 0.2738, OR: 0.7579, 95% CI: 0.2513 to 2.2350) (**Table 1**).

### The increased severity of anxiety and lower quality of life in IBD patients during the COVID-19 pandemic

Thirty-two IBD patients and 44 healthy controls were recruited to evaluate the psychological condition and quality of life of IBD patients during the COVID-19 pandemic; there was no significant difference regarding age and gender (gender: p=0.4630, χ^2^ = 0.5387, OR: 0.7101, 95% CI: 0.2897 to 1.6990). According to the GAD-7, the prevalence rate of anxiety disorder in IBD patients was significantly higher than in healthy controls (p=0.0027, χ^2^ = 8.9890, OR: 4.7900, 95% CI: 1.6100 to 12.8100) (**Supplemental Table 3**), and the GAD-7 scores of IBD patients was also obviously higher than that in healthy controls (**Supplementary Figure 1A**, Mann-Whitney U test, p=0.0031). We further assessed the quality of life of the IBD patients using the GIQLI. Patients with higher anxiety scores had lower GIQLI scores (**Table 2**). Pearson’s correlation analysis revealed a negative correlation between the GAD-7 scores and the GIQLI scores (r2=0.3535, p<0.0010) (**Supplementary Figure 1B**).

**Table 2:** GAD-7 and GIQLI scores in patients with IBD.

Groups (GAD-7 score)	Number (%)	GIQLI score (mean ± SD)
**No anxiety (≤4)**	17 (53.1250%)	86.1600 ± 2.9250
**Mild anxiety (≥5)**	12 (37.5000%)	77.8800 ± 2.6560
**Moderate or severe anxiety(≥10)**	3 (9.3750%)	55.3900 ± 12.2500
**Total**	32	80.1700 ± 2.5940

### DSS-induced colitis model showing obvious anxious behaviors

We established a comorbid mouse model of DSS-induced colitis and depression or anxiety-related behaviors. The experimental mice were administered 2.5% DSS for three days, then the depression or anxiety-related behaviors were evaluated (**Supplementary Figure 2A**); we found that there was no decrease in preference for 1% sucrose in the DSS group compared with the control group in the SPT (**Supplementary Figure 2B**, t(13)=0.9002, p=0.3844). The DSS mice showed no change in the time spent in the open arm (TSOA) and the number of entries into the open arm (NEOA), and increased the number of entries into each arm (NEEA) compared with the control group in the EPM (**Supplementary Figure 2C**, TSOA: Mann Whitney test, p=0.9551; NEOA: t(13)=1.1030, p=0.2889; NEEA: Mann Whitney test, p=0.0103). Meanwhile, there was no difference in the latency of movement toward the illuminated compartment (LIC), the time spent in the illuminated compartment (TSIC), and the counts between illuminated and dark compartments (CIDC) between groups (**Supplementary Figure 2D**, LIC: t(13)=0.08467, p=0.9338; TSIC: t(13)=1.1470, p=0.2719; CIDC: t(13)=1.5530, p=0.1444). There was no alteration in OFT, showing nearly the same time spent in the central zone (TSCZ), total distance in the central zone (TDCZ), and the total distance traveled (TDT) (**Supplementary Figure 2E**, TSCZ: t(13)=0.7111, p=0.4896; TDCZ: t(13)=0.9131, p=0.3778; TDT: t(13)=0.4609, p=0.6525). Moreover, the locomotor activity was evaluated to exclude non-specific motor activity, and the results indicated that there was no comparable difference between the groups (**Supplementary Figure 2F**, treatment: F(1,13)=0.0135, p=0.9093; timepoints: F(14,182)=20.8000, p<0.0010; treatment × timepoints: F(14,182)=0.7626, p=0.7085, Total distance: Mann Whitney test, p=0.6943). Finally, compared with the control group, the DSS mice showed increased DAI (p=0.0010 and p<0.0010) (**Supplementary Figure 2G**, treatment: F(1,13)=32.9900, p<0.0010; timepoints: F(3,39)=11.6400, p<0.0010; treatment × timepoints: F(3,39)=9.7650, p<0.0010), but there was still no difference in visceral sensitivity (VS) (**Supplementary Figure 2H**, t(13)=0.5136, p=0.6162) and the colonic length (CL) (**Supplementary Figure 2I**, t(13)=0.2725, p=0.7895).

**Figure 1:**
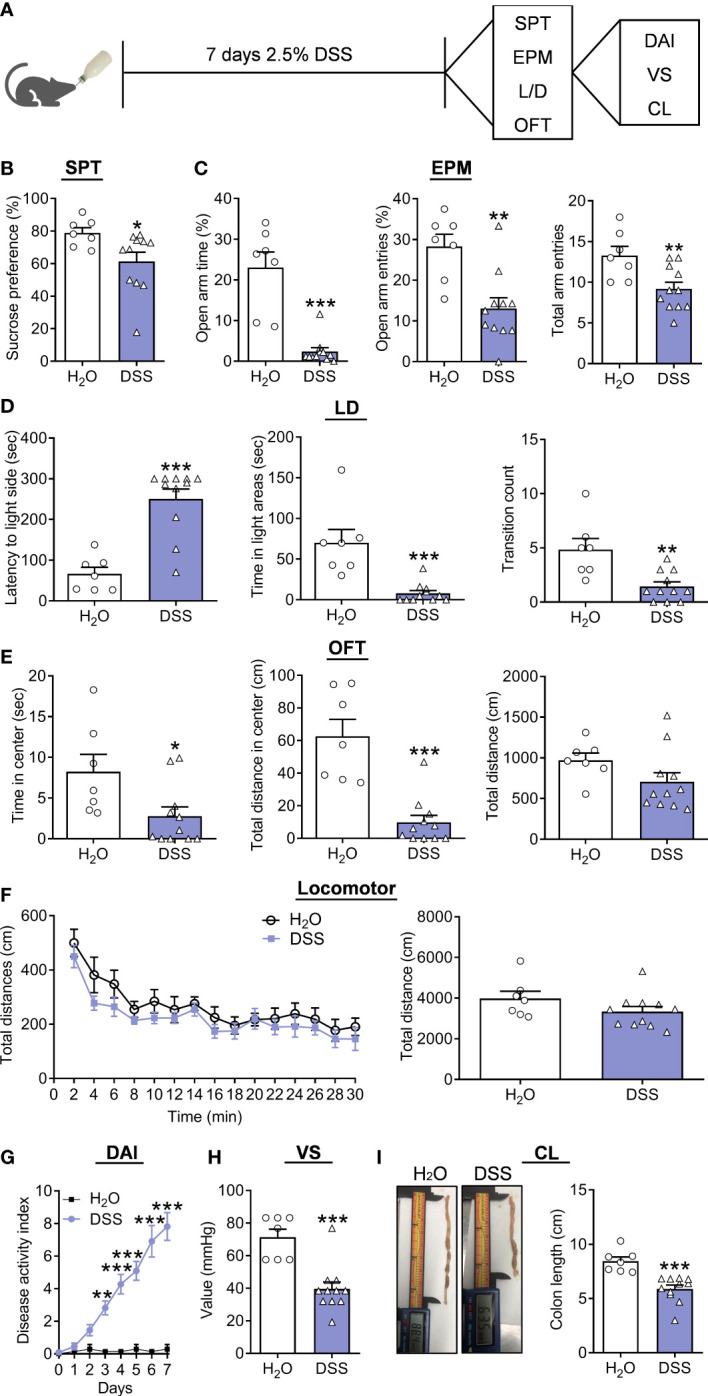
Depression/anxiety-related behaviors and colitis induced by chronic DSS (7 days) application. **(A)** Schematic diagram of experimental designs. **(B)** Sucrose preference test (SPT). **(C)** Elevated plus-maze test (EPM). **(D)** Light-dark (LD). **(E)** Open-field test (OFT). **(F)** Locomotor activity. **(G)** Disease Activity Index (DAI). **(H)** Visceral sensitivity (VS). **(I)** Colonic length (CL). H_2_O group: n= 7, DSS group: n=11. *p < 0.05, **p < 0.01, ***p < 0.001, compared to the control group.

In the following process, another batch of new mice was supplied with 2.5% DSS for seven days (**Figure 1A**). The behavioral testing results demonstrated that DSS-treated mice showed a reduced preference for 1% sucrose compared to controls in SPT (**Figure 1B**, Mann Whitney test, p=0.0268). The decreased TSOA, NEOA, and NEEA were observed of the DSS group in the EPM test (**Figure 1C**, TSOA: Mann Whitney test, p<0.0010; NEOA: t(16)=3.7340, p=0.0018; NEEA: t(16)=3.0120, p=0.0083). In the LD test, the DSS group showed increased LIC, decreased TSIC and CIDC (**Figure 1D**, LIC: Mann Whitney test, p<0.0010; TSIC: Mann Whitney test, p<0.0010; CIDC: t(16)=3.5870, p=0.0025). DSS group also had decreased TSCZ and decreased tendency for TDCZ (**Figure 1E**, TSCZ: Mann Whitney test, p=0.0187; TDCZ: Mann Whitney test, p<0.0010; TDT: Mann Whitney test, p=0.0693) in the OFT. In the end, we measured locomotor activity in these mice, and found that there was no difference between groups (**Figure 1F**, treatment: F(6, 16)=6.3090, p<0.0010; timepoints: F(14, 224)=13.2300, p<0.0010; treatment × timepoints: F(14, 224)=0.3570, p=0.9848; Total distance: t(16) =1.5260, p=0.1465). Furthermore, we observed that the DAI in the DSS group was increased (**Figure 1G**, treatment: F(16, 112)=5.2770, p<0.0010; timepoints: F(7, 112) =23.4900, p<0.0010; treatment × timepoints: F(7, 112)=21.9400, p<0.0010), and VS was also elevated in the DSS group (**Figure 1H**, t(16)=4.7550, p=0.0002). The DSS group’s CL was significantly shorter than that of the control group (**Figure 1I**, t(16)=4.6840, p=0.0002).

Moreover, correlation analysis indicated that the percent of sucrose was positively correlated with VS of colitis (**Supplementary Figure 3A**), and the TSOA, NEOA, and NEEA were also positively correlated with VS of colitis (**Supplementary Figure 3B**). LIC were negatively correlated with VS, while TSIC and CIDC were positively correlated (**Supplementary Figure 3C**). Moreover, the positive correlation of TSCZ and TDCZ with VS was revealed in the DSS group (**Supplementary Figure 3D**). At the same time, we observed the percent of sucrose positively correlated with CL in colitis (**Supplementary Figure 4A**), and the TSOA, NEOA, and NEEA positively correlated with CL in colitis (**Supplementary Figure 4B**). Negative correlation was found with LIC and CL, while TSIC and CIDC were positively correlated (**Supplementary Figure 4C**). Finally, a tendency for positive correlation between TDCZ and CL was revealed in the DSS group (**Supplementary Figure 4D**).

### The DSS treatment accelerated the anxiety-related behaviors and symptoms of colitis induced by subthreshold chronic stress

**Figure 2:**
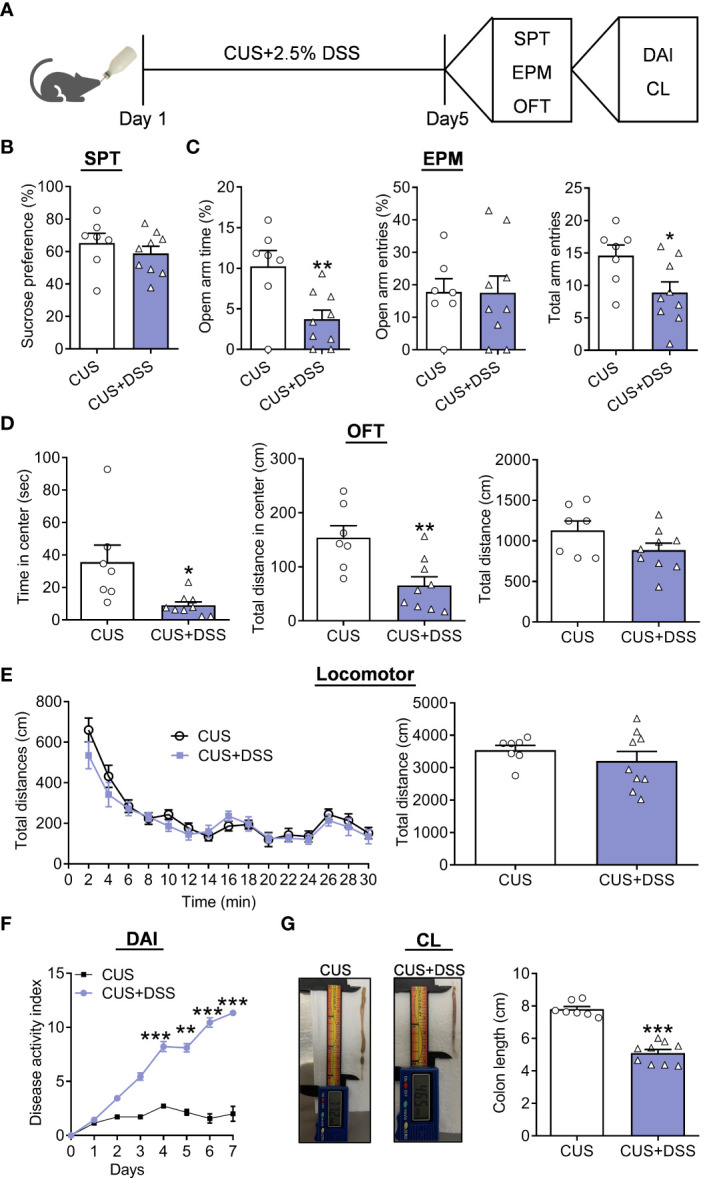
Depression/anxiety-related behaviors and colitis induced by chronic DSS/stress treatment. **(A)** Schematic diagram of experimental designs. **(B)** Sucrose preference test (SPT). **(C)** Elevated plus-maze test (EPM). **(D)** Open-field test (OFT). **(E)** Locomotor activity. **(F)** Disease Activity Index (DAI). **(G)** Colonic length (CL). H_2_O group: n=7, DSS group: n=9. *p < 0.05, **p < 0.01, ***p < 0.001, compared to the control group.

Our previous reports proved that five days of CUS was a well-established paradigm to evaluate the susceptibility to stress-induced anxiety/depression-related behaviors (21), which was applied to validate whether DSS treatment can affect the course and severity of anxiety. Therefore, we established a mouse model of the CUS and DSS complex application paradigm for five days (**Figure 2A**). The behavioral test results showed that the preference for 1% sucrose remains unchanged in the CUS+DSS group in SPT when compared with the CUS group (**Figure 2B**, t(14)=0.8882, *p*=0.3894). CUS+DSS treated mice showed decreased TSOA and NEEA, and were accompanied by similar NEOA (**Figure 2C**, TSOA: t(14)=3.0690, *p*=0.0083; NEOA: t(14)=0.0409, *p*=0.9679; NEEA: t(14)=2.4020, *p*=0.0308) in the EPM test. Meanwhile, in OFT, the decreased TSCZ and TDCZ were found in the CUS+DSS group, while leaving the TDT unchanged (**Figure 2D**, TSCZ: Unpaired t-test with Welch’s correction, *p*=0.0441; TDCZ: t(14)=3.3180, *p*=0.0051; TDT: t(14)=1.7010, *p*=0.1110). Finally, we observed that there was no difference in the locomotor activity between the groups (**Figure 2E**, treatment: F(1,14)=0.8369, *p*=0.3758; timepoints: F(14,196) =29.9500, *p*<0.0010; treatment × timepoints: F(14,196) =0.9275, *p*=0.5302; t(14)=0.9148, *p*=0.3758). Furthermore, the symptoms of colitis were also tested, and compared with the CUS group, DAI in the CUS+DSS group was significantly increased (**Figure 2F**, treatment: F(1,14)=216.3000, *p*<0.0010; timepoints: F(7,98)=154.2000, *p*<0.0010; treatment × timepoints: F(7,98)=88.7100, *p*<0.0010). The length of the colon were significantly shortened in the CUS+DSS group compared with the CUS group (**Figure 2G**, t(14)=9.0840, *p*<0.0010).

**Figure 3:**
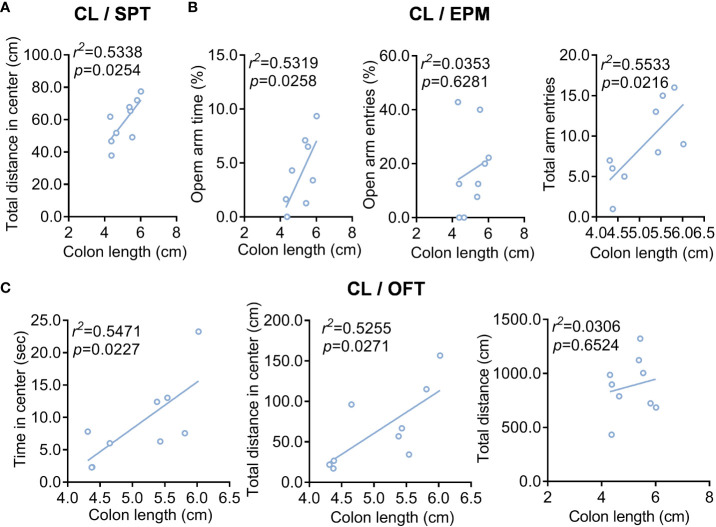
The correlation analysis of colonic length with depression/anxiety-related behaviors in the DSS/stress-treated mice. **(A)** Colonic length (CL)/SPT. **(B)** Colonic length (CL)/EPM. **(C)** Colonic length (CL)/OFT. n=9.

Furthermore, we also observed the percentage of sucrose positively correlates with CL in colitis (**Figure 3A**). Still, the TSOA and NEEA positively correlated with CL in colitis (**Figure 3B**). Positive correlation with CL in colitis was found for TSCZ and TDCZ (**Figure 3C**).

### IL-19 was a pivotal player in the DSS/Stress-induced comorbidities in colitis and anxiety

**Figure 4:**
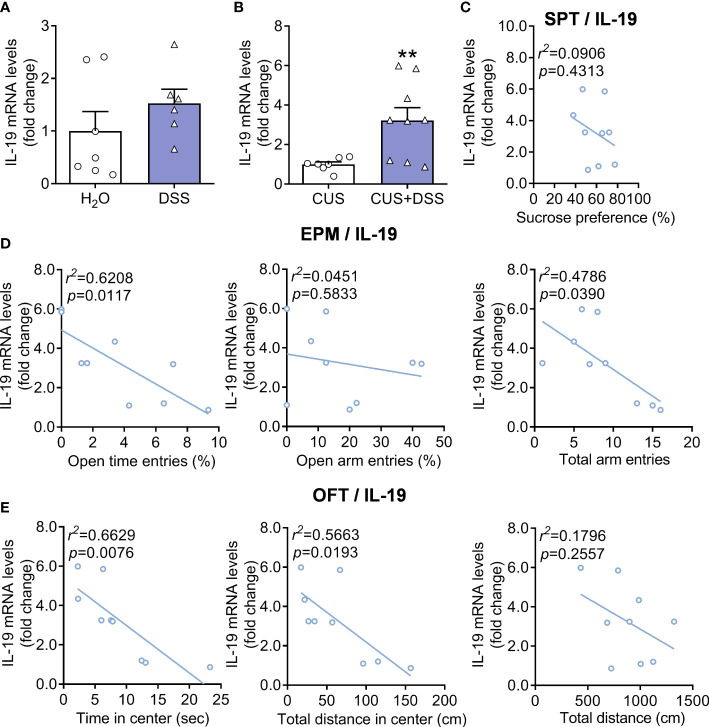
Expression levels of IL-19 in DSS or DSS/CUS mice colon. IL-19 mRNA levels in **(A)** DSS or **(B)** DSS/CUS mice. H_2_O group: n= 7, DSS group: n=6. CUS group: n=7, CUS + DSS group: n=9. The correlation analysis of **(C)** SPT, **(D)** EPM, and **(E)** OFT with the mRNA levels of IL-19 in the DSS/CUS mice. **p < 0.01, compared to the control group.

Further efforts were conducted to explore whether IL-19 was involved in the pathogenesis of colon and anxiety; we measured the levels of IL-19 in colon of only DSS-treated and DSS/CUS models and found that the messenger RNA (mRNA) levels of IL-19 were increased in DSS/CUS treated mice, but not exclusively DSS treated mice (**Figure 4A**, Mann Whitney test, p=0.1807; **Figure 4B**, Unpaired t test with Welch’s correction, p=0.0086). The ongoing correlation analysis indicated that mRNA levels of IL-19 positively correlated with anxiety-related behaviors but not depressive behaviors regarding the sucrose preference test in the DSS/CUS group (**Figures 4C–E**).

### Overexpression of IL-19 in colon produced anxiety-related behaviors

**Figure 5:**
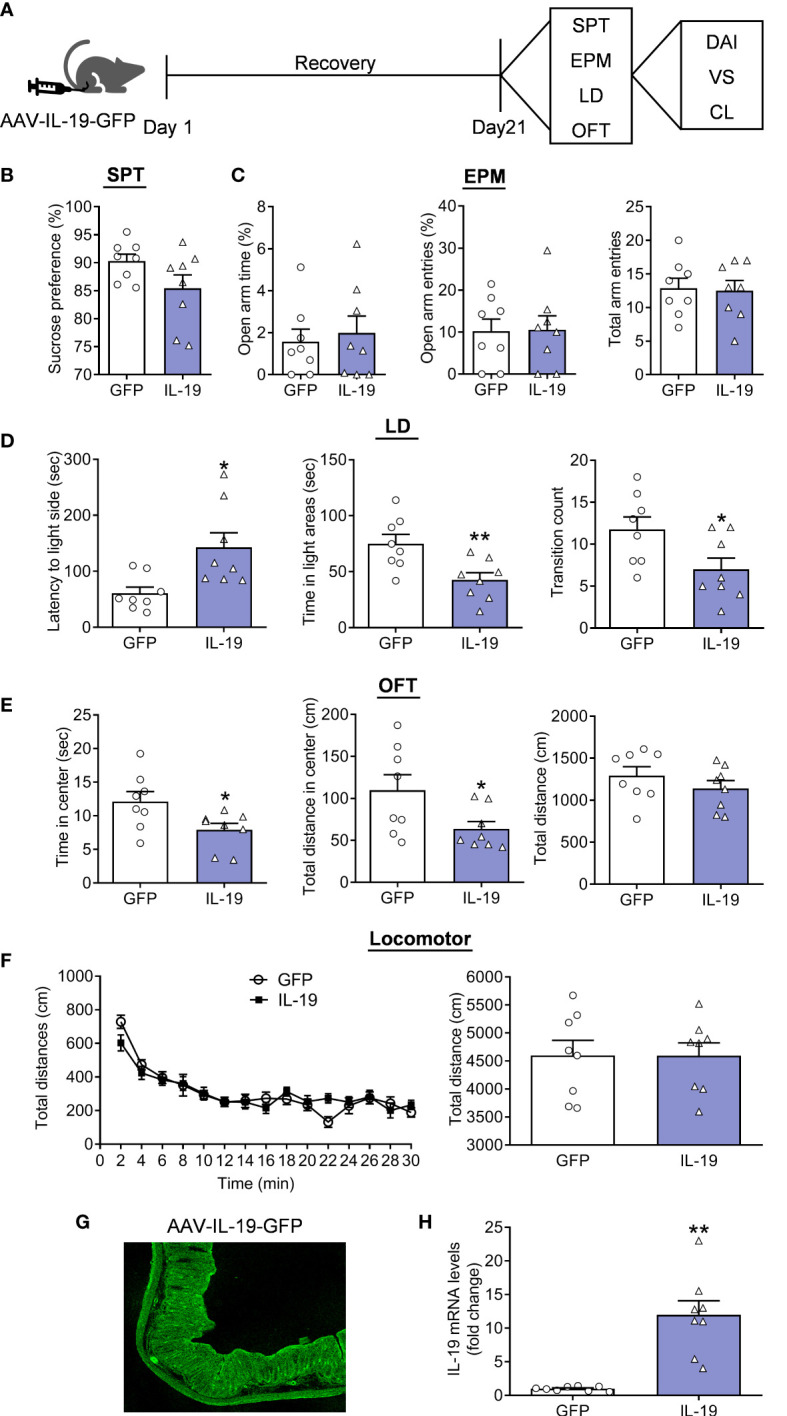
The effect of colonic IL-19 overexpression in depression/anxiety-related behaviors and colitis. **(A)** Schematic diagram of experimental designs. **(B)** Sucrose preference test (SPT). **(C)** Elevated plus-maze test (EPM). **(D)** Light-dark (LD). **(E)** Open-field test (OFT). **(F)** Locomotor activity. **(G)** Representative image showing the expression of AAV-IL-19-GFP. **(H)** The IL-19 mRNA levels in the colon. GFP group: n=8, IL-19 group: n=8. *p < 0.05, **p < 0.01, compared to the control group.

To confirm the cause-effect relation between IL-19 in colitis and anxiety, we overexpressed IL-19 in colitis through an AAV-mediating strategy by colonic perfusion of AAV-IL-19-GFP or AAV-GFP (**Figure 5A**). After a 21-day recovery, the behavioral measurement indicated that there were no changes in preference for 1% sucrose in the AAV-IL-19-GFP group (**Figure 5B**, t(14)=1.8020, p=0.0932). There was no alteration in the anxiety-related behaviors in the EPM test, with nearly the same TSOA, NEOA, and NEEA (**Figure 5C**, TSOA: t(14)=0.4201, p=0.6808; NEOA: t(14)=0.0701, p=0.9451; NEEA: t(14)=0.1772, p=0.8619). The LD test showed an increased LIC, decreased TSIC and CIDC in the AAV-IL-19-GFP group (**Figure 5D**, LIC: Mann Whitney test, p=0.0104; TSIC: t(14)=3.1140, p=0.0076; CIDC: t(14)=2.3570, p=0.0335) comparing to the control group. Furthermore, the AAV-IL-19-GFP group also displayed a decreased TSCZ and TDCZ, and no change in TDT (**Figure 5E**, TSCZ: t(14)=2.3980, p=0.0310; TDCZ: Mann Whitney test, p=0.0379; TDT: t(14)=1.0830, p=0.2971) in OFT. Moreover, the locomotor activity was evaluated to indicate that there was no significant difference between the groups (**Figure 5F**, treatment: F(4, 14)=0.0006, p=0.9980; timepoints: F(14,196)=25.5100, p<0.0010; treatment × timepoints: F(14, 196)=1.5080, p=0.1107; t(14)=0.0026, p=0.9980). Furthermore, the expression specificity was demonstrated by the GFP fluoresce expression in colonic tissue, which proved that the AAV-IL-19-GFP virus was infected successfully (**Figure 5G**). The IL-19 mRNA levels in the colonic were also higher in the AAV-IL-19-GFP group than in the GFP group (**Figure 5H**, unpaired t-test with Welch’s correction, p=0.0012).

### Overexpression of IL-19 in colon accelerates the progression of DSS/Stress-induced comorbidities of colitis and anxiety

**Figure 6:**
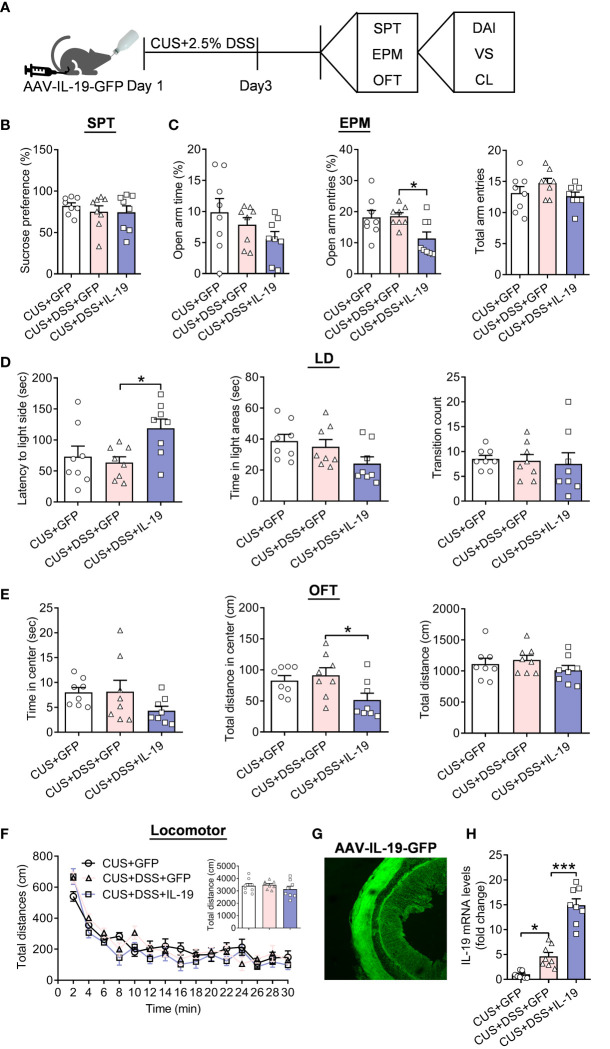
The effect of colonic IL-19 overexpression in depression/anxiety-related behaviors and colitis in the DSS/CUS treated mice. **(A)** Schematic diagram of experimental designs. **(B)** Sucrose preference test (SPT). **(C)** Elevated plus-maze test (EPM). **(D)** Light-dark (LD). **(E)** Open-field test (OFT). **(F)** Locomotor activity. **(G)** Representative image showing the expression of AAV-IL-19-GFP. **(H)** The IL-19 mRNA levels in the colon. CUS+GFP group: n=8, CUS+DSS+GFP group: n=8, CUS+DSS+IL-19 group: n=8. *p < 0.05, ***p < 0.01, compared to the control group.

Next, we aimed to identify the effect of IL-19 in affecting anxiety-related behaviors in the DSS/CUS-induced comorbidities of colitis and anxiety model; the AAV-IL-19-GFP or AAV-GFP mice were given the CUS/DSS treatment with a shortened period (three days) to explore the role of IL-19 in the time window of progression of DSS/CUS-induced comorbidities of colitis and anxiety (**Figure 6A**). We found that the preference for 1% sucrose in the three groups remains unchanged for the CUS/DSS (p=0.7172) or AAV-IL-19-GFP treatment (p=0.9964) (**Figure 6B**, F(2,21)=0.4554, p=0.6403). EPM test showed that the only CUS/DSS treatment did not alter the TSOA (p>0.9999), NEOA (p=0.9902), and NEEA (p=0.3743), while AAV-IL-19-GFP treatment reduced the NEOA (p=0.0357), but not TSOA (p=0.6880) and NEEA (p=0.1969) (**Figure 6C**, TSOA: Kruskal-Wallis test, p=0.1959; NEOA: F(2,21)=4.5690, p=0.0225; NEEA: F(2,21)=1.7540, p=0.1976). The LD test showed that the LIC, TSIC, and CIDC were not changed by the CUS/DSS treatment (p=0.8861; p=0.8323; p=0.9843), but AAV-IL-19-GFP treatment enhanced the LIC (p=0.0315; p=0.2201; p=0.9570) (**Figure 6D**, LIC: F(2,21)=4.2960, p=0.0273; TSIC: F(2,21)=2.8710, p=0.0790; CIDC: F(2,21)=0.1044, p=0.9014). Meanwhile, in the OFT, we observed that AAV-IL-19-GFP treatment decreased the TDCZ (p=0.0486), but not TSCZ (p=0.2074) and TDT (p=0.3445) after CUS/DSS treatment (**Figure 6E**, TSCZ: F(2,21)=2.002, p=0.1600; TDCZ: Kruskal-Wallis test, p=0.0424; TDT :F(2,21)=1.0390, p=0.3713). The locomotor activity results indicated that there was no significant difference among the groups (**Figure 6F**, treatment: F (2, 21)=2.0980, p<0.0010; timepoints: F (14, 294)=43.0100, p<0.0010; treatment × timepoints: F(28, 294)=1.4490, p=0.0710; F(2,21)=0.8881, p=0.4263). Furthermore, the expression of GFP fluorescent in the colon was revealed to validate the successful infection of the AAV-IL-19-GFP virus (**Figure 6G**). In the end, IL-19 mRNA levels in the colon were measured. The results indicated that IL-19 mRNA levels were elevated by the CUS/DSS treatment (p=0.0201), while AAV-IL-19-GFP induced a dramatic increase of IL-19 in the colitis (p<0.0010) (**Figure 6H**, F(2,21)=68.3000, p<0.0010).

**Figure 7:**
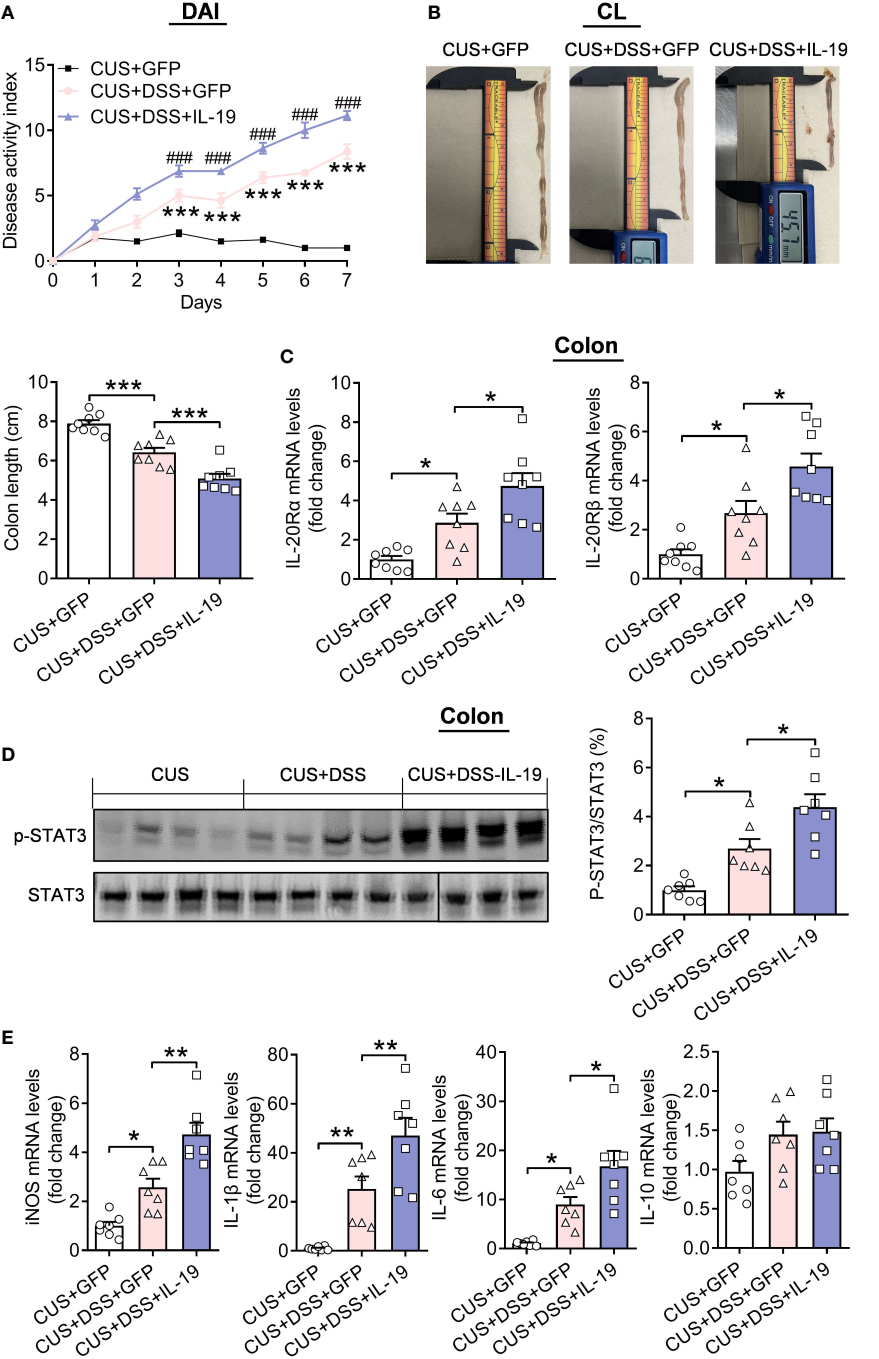
The effect of colonic IL-19 overexpression in the colitis in the DSS/CUS treated mice. **(A)** Disease Activity Index (DAI). **(B)** Colonic length (CL). **(C)** IL-20Ra and IL-20Rb mRNA levels in the colon. **(D)** p-STAT3 levels in the colon. CUS + GFP group: n=8, CUS+DSS+GFP group: n=8, CUS+DSS+IL-19 group: n=8. **(E)** The mRNA levels of iNOS, IL-1β, IL-6, and IL-10 in the colon. CUS + GFP group: n=7, CUS+DSS+GFP group: n=7, CUS+DSS+IL-19 group: n=7. *p < 0.05, **p < 0.01, ***p < 0.001, ****p < 0.001, compared to the control group.

Furthermore, AAV-IL-19-GFP treatment accelerates the severity of colitis through increased DAI (**Figure 7A**, treatment: F(2,21)=105.6000, p<0.0010; timepoints: F(7,147)=223.3000, p<0.0010; treatment × timepoints: F(14,147)=52.0800, p<0.0010) and decreased colonic length (p<0.0010) (**Figure 7B**, F(2,21)=40.6300, p<0.0010). We tested the underlying pathways of IL-19 in the colon. The mRNA levels of IL-19 receptors: IL-20Rα and IL-20Rβ were up-regulated by CUS/DSS treatment (p=0.0299; p=0.0293), and IL-19 overexpression aggravated their increased expression (p=0.0301; p=0.0136). (**Figure 7C**, IL-20Rα: F(2,21)=15.2800, p<0.0010; IL-20Rβ: F(2,21)=17.4200, p<0.0010). It was reported that STAT3 was the key molecular pathway of IL-19 (26, 27). Therefore, we found IL-19 overexpression strengthened the increased phosphorylation levels of STAT3 induced by CUS/DSS treatment (p=0.0168; p=0.0174) (**Figure 7D**, F(2, 18)=18.9000, p<0.0010).

Moreover, the mRNA expressions of inducible nitric oxide synthase (iNOS), IL-1β, IL-6, and IL-10 in the colon were measured, and it found that the mRNA expressions of iNOS, IL-1β, and IL-6 were increased by CUS/DSS treatment (p=0.0146; p=0.0074; p=0.0328), and the overexpression of IL-19 accelerated their expression (p=0.0011; p=0.0076; p=0.0351), while the mRNA expression of IL-10 remained unchanged among the groups (p=0.1725; p=0.6539; p=0.0847) (**Figure 7E**, iNOS: F(2,18)=28.1500, p<0.0010; IL-1β: F(2,18)=20.1000, p<0.0010; IL-6: F(2,18)=15.0700, p<0.0010; IL-10: F(2, 18)=3.2330, p=0.0635).

### IL-19 inhibited the expression of BDNF in the hippocampus by suppressing ERK/CREB pathway

**Figure 8:**
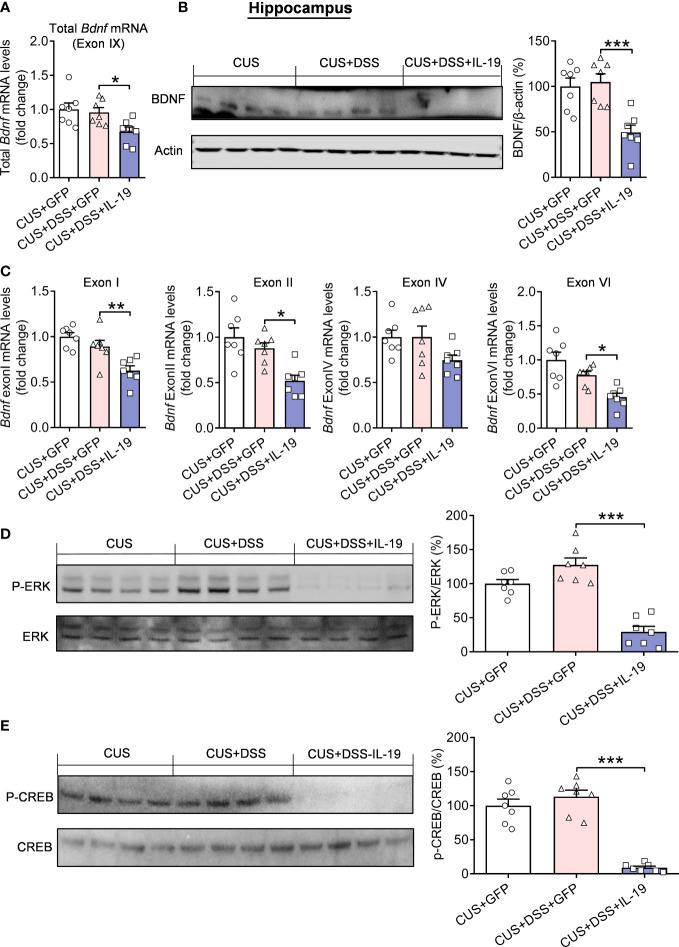
Regulation of hippocampal ERK-CREB-BDNF pathway by colonic IL-19 overexpression in the DSS/CUS treated mice. **(A)** The mRNA and **(B)** protein levels of BDNF in the hippocampus. **(C)** The mRNA levels of BDNF-specific exons. **(D)** p-ERK and p-CREB levels in the hippocampus. CUS + GFP group: n=7, CUS+DSS+GFP group: n=7, CUS+DSS+IL-19 group: n=7. *p < 0.05, **p < 0.001, ***p < 0.001, compared to the control group.

Further research was focused on the traditional ERK/CREB/BDNF signal transduction pathway, which has been reported to be related to the pathogenesis of anxiety (25, 28, 29). Therefore, to investigate the molecular mechanism that mediates the produced anxiety-related behaviors, we first measured the mRNA and protein expression levels of BDNF. The results indicated that overexpression of IL-19 decreased the total mRNA expression (*p*=0.0465) and protein (*p*<0.0010) of BDNF (**Figure 8A**, F(2,18)=5.2450, *p*=0.0160; **Figure 8B**, F(2,18) = 12.2300, *p*<0.0010) in the CUS/DSS treated mice, and its corresponding spliced exons detection results revealed that the expression of BDNF exon I (*p*=0.0087), II (*p*=0.0115) and VI (*p*=0.0265) was remarkably decreased by overexpression of IL-19, leaving exon IV (*p*=0.1280) unchanged (**Figure 8C**, BDNF exon I: F(2,18)=12.1000, *p*<0.0010; BDNF exon II: F(2,18)=10.3600, *p*=0.0010; BDNF exon IV: F(2,18)=2.7980, *p*=0.0875; BDNF exon VI: F(2,18)=11.9100, *p*<0.0010). Furthermore, we verified the IL-19 overexpression significantly reduced the expression of p-ERK (*p*<0.0010) and p-CREB (*p*<0.0010) in the hippocampus of CUS/DSS treated mice (**Figure 8D**, p-ERK: F(2,18)=38.3100, *p*<0.0010; **Figure 8E**, p-CREB: F(2,18)=51.7400, *p*<0.0010).

## Discussion

Many clinical studies have shown the co-existence and co-interaction of IBD and anxiety (30, 31). The present research examined the increased incidence rate of IBD after the epidemic of COVID-19, with higher anxious levels and poorer gastrointestinal quality of life. We found that the DSS/CUS-induced comorbid model of colitis and anxiety showed up-regulated expression of IL-19 in the colon, and overexpression of IL-19 in the colon produced anxiety-related behaviors, and enhanced the sensitivity to the DSS/CUS-induced anxiety and symptoms of colitis. Moreover, overexpression of IL-19 in the colon also accelerated the expression of pro-inflammatory factors (iNOS, IL-1β, and IL-6), activated the STAT3 signaling pathway in the colon, and reduced the expression levels of BDNF and the activity of ERK and CREB signaling pathway in the hippocampus.

IBD affects the quality of life of patients with prolonged disease, hematochezia, abdominal pain, and other obvious clinical symptoms, but it also causes psychological problems such as anxiety and depression in patients (4, 5). We investigated the hospitalization rate of ulcerative colitis during the COVID-19 pandemic, and the incidence was increased significantly, which may be related to the unknown situation of the new virus. On the other hand, it shows that anxiety or depression also affects intestinal symptoms and aggravates the disease (32). It has been reported that the probability of recurrence of IBD is increased over two times when patients face perceived stress (33, 34). In addition, the symptoms of depression and anxiety are related to the disease activity of IBD (35). The intestine and the brain are intimately connected by the gut-brain axis, a complex bidirectional system, in which the central and enteric nervous systems communicate involving endocrine, immune and neuronal pathways (8, 36), indicating the interactional characters between colitis and anxiety, and there was one report indicating that CUS promotes DSS induced colitis with increased and prolonged DAI scores (37), while, our study identified that colitis condition aggravates the symptoms of anxiety. Furthermore, in our study, we focused on the inflammatory factor IL-19, and found that the expression levels of IL-19 was dramatically increased in the colon of the DSS/CUS model, which was matched to the finding that the expression of IL-19 mRNA in the colonic mucosa of patients with active IBD is increased (14), meanwhile, it was reported that DSS administration for five days revealed that the expression of IL-19 mRNA was induced in the distal colon in wild-type mice (38). Unexpectedly, we found that the expression of IL-19 mRNA in the colon of single DSS induced model remained unchanged. These results validated that IL-19 was crucial in IBD’s pathological process. This diverse expression profile of IL-19 in these two animal models may be due to the additional CUS; this implies that even though the emerging phenotypes seem nearly the same in the two models, the underlying molecular mechanisms may be disparate.

In order to identify the exact effect of IL-19 in the comorbidities due to colitis and anxiety, we infected the intestinal tract with the IL-19 overexpression virus. We found that simple IL-19 overexpression can produce anxiety-related behaviors and accelerate the DSS/CUS-induced colitis and anxiety phenomenons. However, on the contrary, previous results indicated that the IL-19−/− mice were highly susceptible to DSS-induced colitis, resulting in severe weight loss and death (38, 39). As a member of the IL-10 family, it is generally believed to have a protective effect (38, 40), and some studies showed a pro-inflammatory effect in some cases (15). All this evidence implied that the role of IL-19 in colitis is controversial; this may be probably due to the side effects or compensatory reactions of the whole immune system or immune cells after the none-specific knockout of IL-19, and the complicated molecular interactions with other genes, such as IL-19 is highly homologous to IL-20 and IL-24, and shares a receptor that is formed by a heterodimer of the IL-20Ra and IL-20Rb subunits to activate the signal transducers and activators of transcription pathways (41, 42), this fact was validated by our results showing IL-19 activated the STAT3 pathways. Moreover, it has been proposed that IL-19 secretion can drive an autocrine feedback loop that amplifies the local expression of cytokines such as IL-1β, IL-6, IL-12, and tumor necrosis factor-α (38), which is in line with our findings that IL-19 overexpression elevated the expression level of IL-1β, IL-6, and iNOS to promote the inflammatory responses. Meanwhile, it was interesting to find the levels of IL-10, which is an anti-inflammatory cytokine, showed an elevation tendency of both CUS+DSS and CUS+DSS+IL-19 groups, even though the similar results was also revealed in same previous reports (43–45). The sexual difference of the morbidity for anxiety was significant (46, 47), neverthless, there is no obvious gender difference in the morbidity of comorbidities of colitis and anxiety, which was in line with our clinical analyzed data, but this phenomenon may be still controversial (48). Therefore, we were not concentrated on the sexual difference in our experimental reseaches, even though there may be potential diversities in the phenotypic and molecular levels (48, 49). However, the concrete relations of these molecules still need further determination.

Subsequently, we explored the potential mechanisms in the brain that mediating anxious behaviors after manipulating IL-19 in the colon. The concentrated focus was moved to the BDNF, a well-characterized pathological gene of anxiety (28, 50). Previous research reported that the levers of BDNF and p-CREB were decreased in the medial prefrontal cortex of comorbidity model with characteristics of both depression and chronic pain induced by DSS and CUS or single DSS (37, 51). Our results demonstrated that IL-19 overexpression suppressed hippocampal BDNF with the specific regulation of exon I, II, and VI. To our knowledge, this is the first research to reveal the regulatory effect between IL-19 and BDNF. Furthermore, we explored the signaling pathway under the manipulation of BDNF expression. We found that IL-19 activated the classical ERK-CREB pathway that adjusts the levels of BDNF in the hippocampus (52, 53). After we proved the effect of peripheric IL-19 in the colon in the activity of the ERK-CREB-BDNF pathway, another question was raised about the role of IL-19 in the hippocampus in the anxiety-related behaviors and potential signaling pathway; in our research, we observed that the mRNA levels of IL-19 in the hippocampus were not significantly changed in the DSS/CUS model (data not shown), indicating hippocampal IL-19 may not be extensively involved in the DSS/CUS induced anxiety behaviors. Nevertheless, the role of peripheric IL-19 in the cerebrocentric activity was direct or indirect effect still remains unclear, because the colonic IL-19 overexpression may flow into brain through the blood-brain barrier (54), and directly activate IL-20R and downstream signaling pathways through blood circulation; meanwhile, our results also showed that colonic IL-19 manipulation also regulate other inflammatory cytokines, which may also exerts effects on the brain functions. Furthermore, the inflammatory reactions induced by colonic IL-19 manipulation may disturb of the permeability of the blood-brain barrier (BBB) to allow the inflow of many neurotoxins (55, 56), which may also have the potentiality to affect the ERK-CREB-BDNF pathway. Still, its potential role in anxiety cannot be excluded, and further research should address this question.

In conclusion, IL-19 plays an essential role in comorbidities related to IBD and anxiety, which can not only control colonic inflammation through the activation of STAT3 but also cause anxiety by down-regulating the ERK-CREB-BDNF pathway. IL-19 and its receptor may be important in designing therapeutic agents for comorbidities related to IBD and anxiety.

## Data availability statement

The original contributions presented in the study are included in the article/**Supplementary Material**. Further inquiries can be directed to the corresponding authors.

## Ethics statement

The studies involving human participants were reviewed and approved by Local Ethics Committee of Binzhou Medical University Hospital. The patients/participants provided their written informed consent to participate in this study. The animal study was reviewed and approved by Local Ethics Committee of Binzhou Medical University Hospital.

## Author contributions

QYL, FTM, XXM, ZS: Conceived and designed the study. JJD, JL, DL, PJC, RXX, DZ, WTW, DW, CLL: Analysis and interpretation of data. CL, HFL, FXW: Wrote the paper, and all authors reviewed the final version of the paper. CL supervised all the experiments. All authors contributed to the article and approved the submitted version.

## Supplementary material

Supplementary Figure 1 The GAD7 scores and its relation to GIQLI score of IBD patients. **(A)** GAD7 scores. **(B)** Correlation analysis of GAD7 scores and GIQLI score of IBD patients. Control group: n=44, IBD group: n=32. ***p* < 0.01, compared to the control group.

Supplementary Figure 2 Depression/anxiety-related behaviors and colitis induced by chronic DSS (three days) application. **(A)** Schematic diagram of experimental designs. **(B)** Sucrose preference test (SPT). **(C)** Elevated plus-maze test (EPM). **(D)** Light-dark (LD). **(E)** Open-field test (OFT). **(F)** Locomotor activity. **(G)** Disease Activity Index (DAI). **(H)** Visceral sensitivity (VS). **(I)** Colonic length (CL). H_2_O group: n= 7, DSS group: n=8. **p* < 0.05, ***p* < 0.01, ****p* < 0.001, compared to the control group.

Supplementary Figure 3 The correlation analysis of colonic length with depression/anxiety-related behaviors in the chronic DSS (three days) treated mice. **(A)** Visceral sensitivity (VS)/SPT. **(B)** Visceral sensitivity (VS)/EPM. **(C)** Visceral sensitivity (VS)/LD. **(D)** Visceral sensitivity (VS)/OFT. n=11.

Supplementary Figure 4 The correlation analysis of colonic length with depression/anxiety-related behaviors in the chronic DSS (three days) treated mice. **(A)** Colonic length (CL)/SPT. **(B)** Colonic length (CL)/EPM. **(C)** Colonic length (CL)/LD. (D) Colonic length (CL)/OFT. n=11.
