# Supplementary figures and images for: The colonic interleukin-19 aggravates the dextran sodium sulfate/stress-induced comorbidities due to colitis and anxiety
Source: Front Immunol. 2023 Mar 2;14:1153344. doi: 10.3389/fimmu.2023.1153344 (PMC10018752; doi:10.3389/fimmu.2023.1153344)

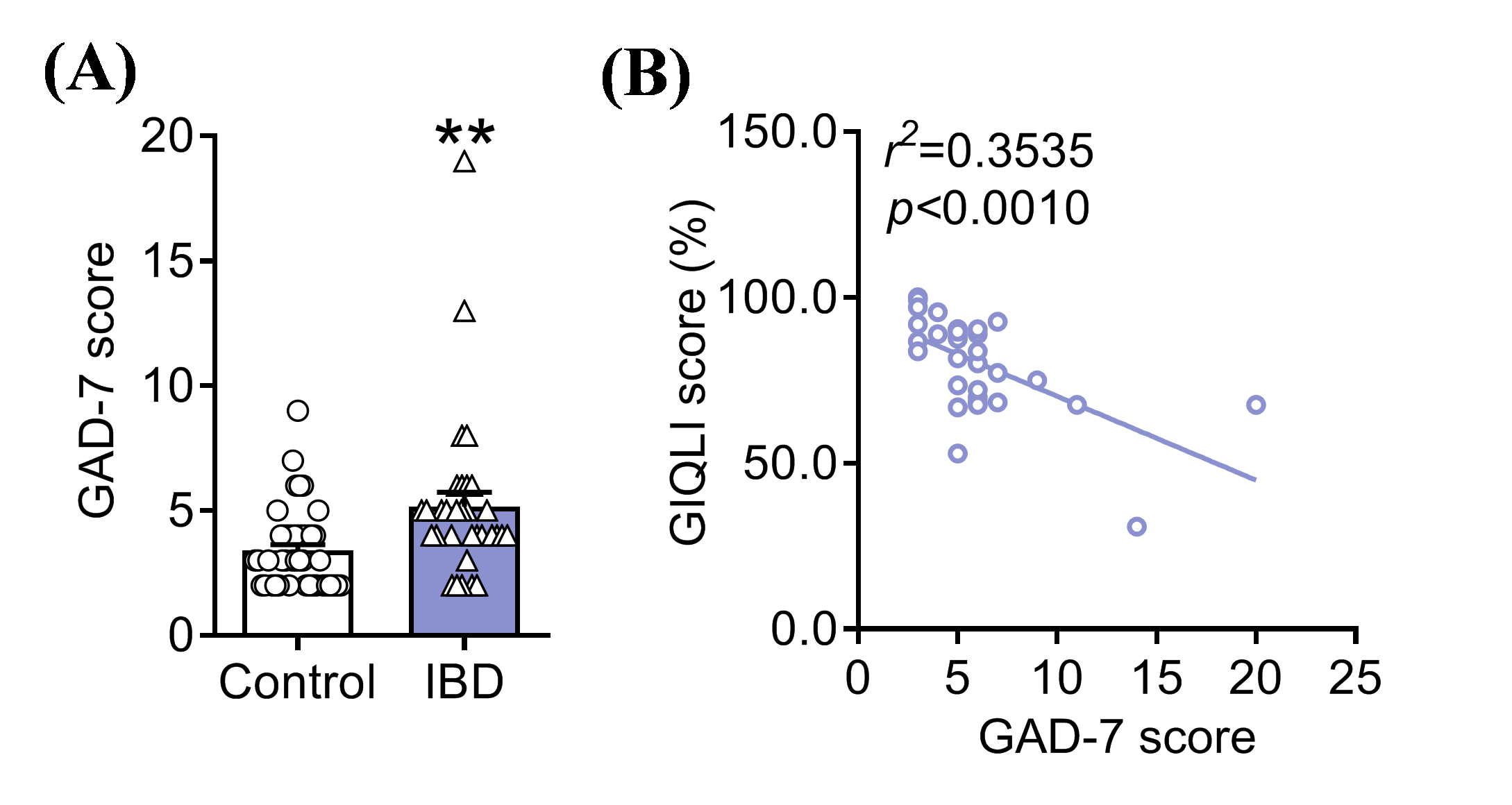

Supplement: Supplementary Figure 1 — The GAD7 scores and its relation to GIQLI score of IBD patients. (A) GAD7 scores. (B) Correlation analysis of GAD7 scores and GIQLI score of IBD patients. Control group: n=44, IBD group: n=32. **p < 0.01, compared to the control group. [file Image_1.tif]

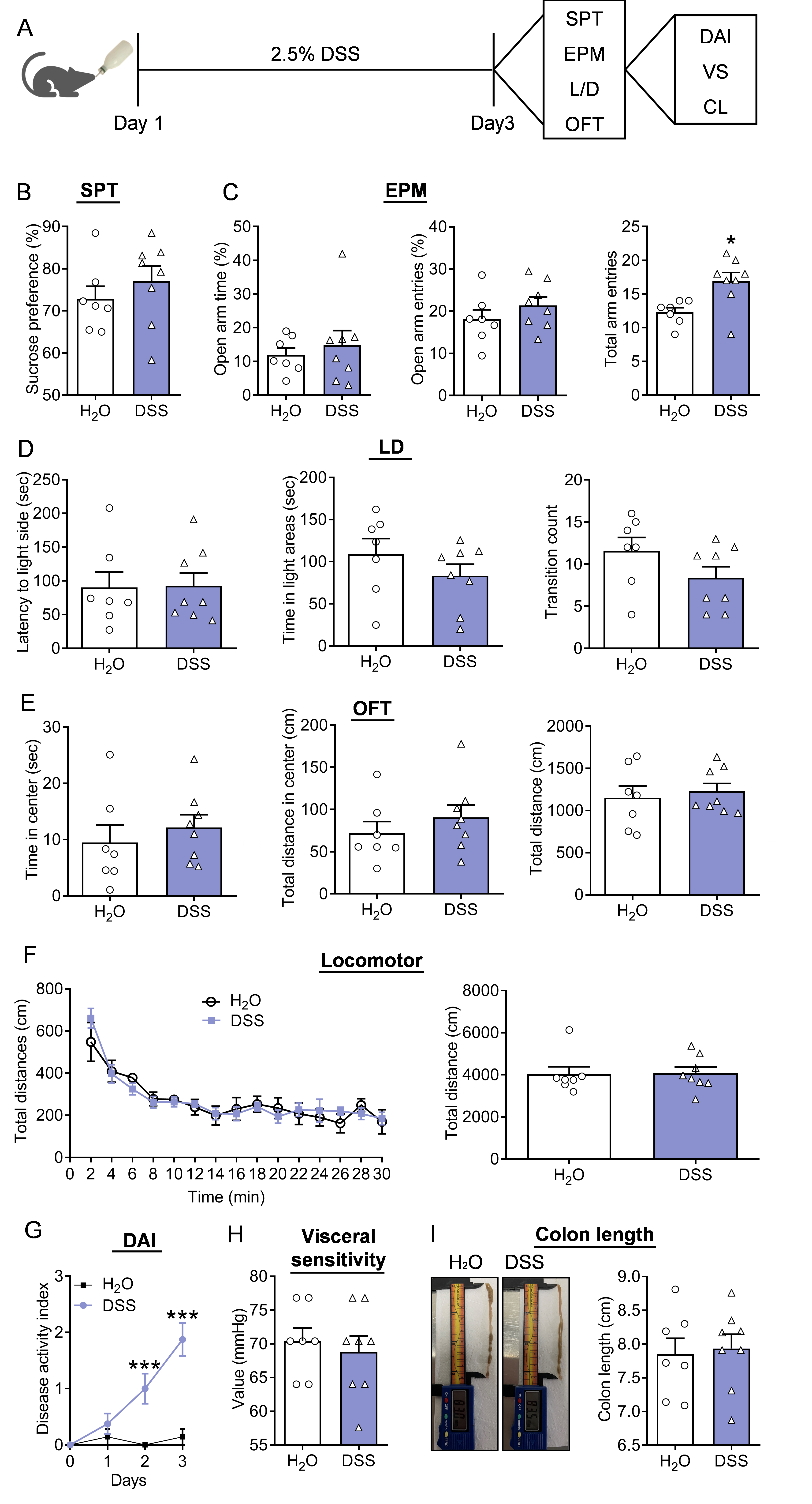

Supplement: Supplementary Figure 2 — Depression/anxiety-related behaviors and colitis induced by chronic DSS (three days) application. (A) Schematic diagram of experimental designs. (B) Sucrose preference test (SPT). (C) Elevated plus-maze test (EPM). (D) Light-dark (LD). (E) Open-field test (OFT). (F) Locomotor activity. (G) Disease Activity Index (DAI). (H) Visceral sensitivity (VS). (I) Colonic length (CL). H2O group: n= 7, DSS group: n=8. *p < 0.05, **p < 0.01, ***p < 0.001, compared to the control group. [file Image_2.tif]

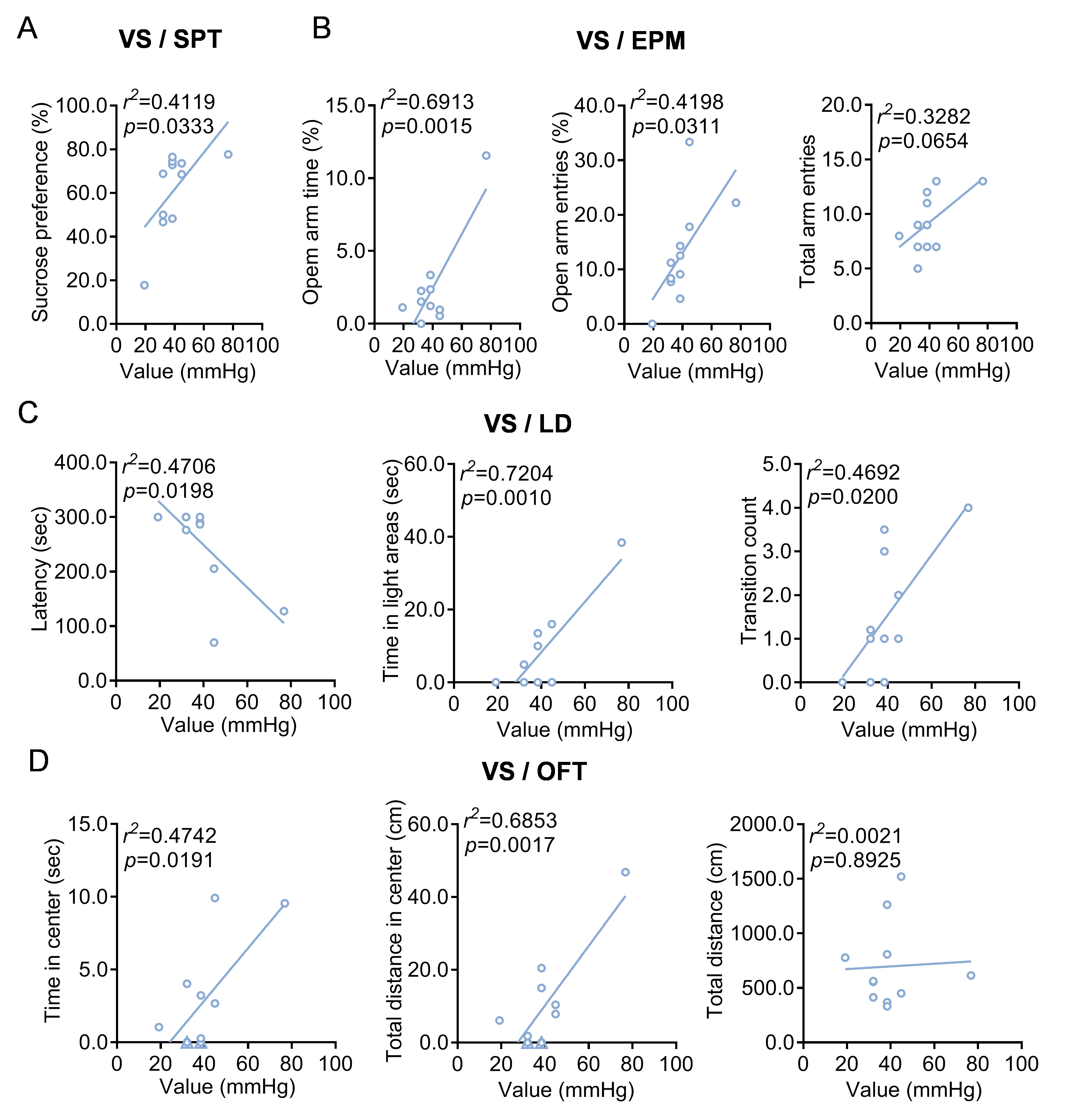

Supplement: Supplementary Figure 3 — The correlation analysis of colonic length with depression/anxiety-related behaviors in the chronic DSS (three days) treated mice. (A) Visceral sensitivity (VS)/SPT. (B) Visceral sensitivity (VS)/EPM. (C) Visceral sensitivity (VS)/LD. (D) Visceral sensitivity (VS)/OFT. n=11. [file Image_3.tif]

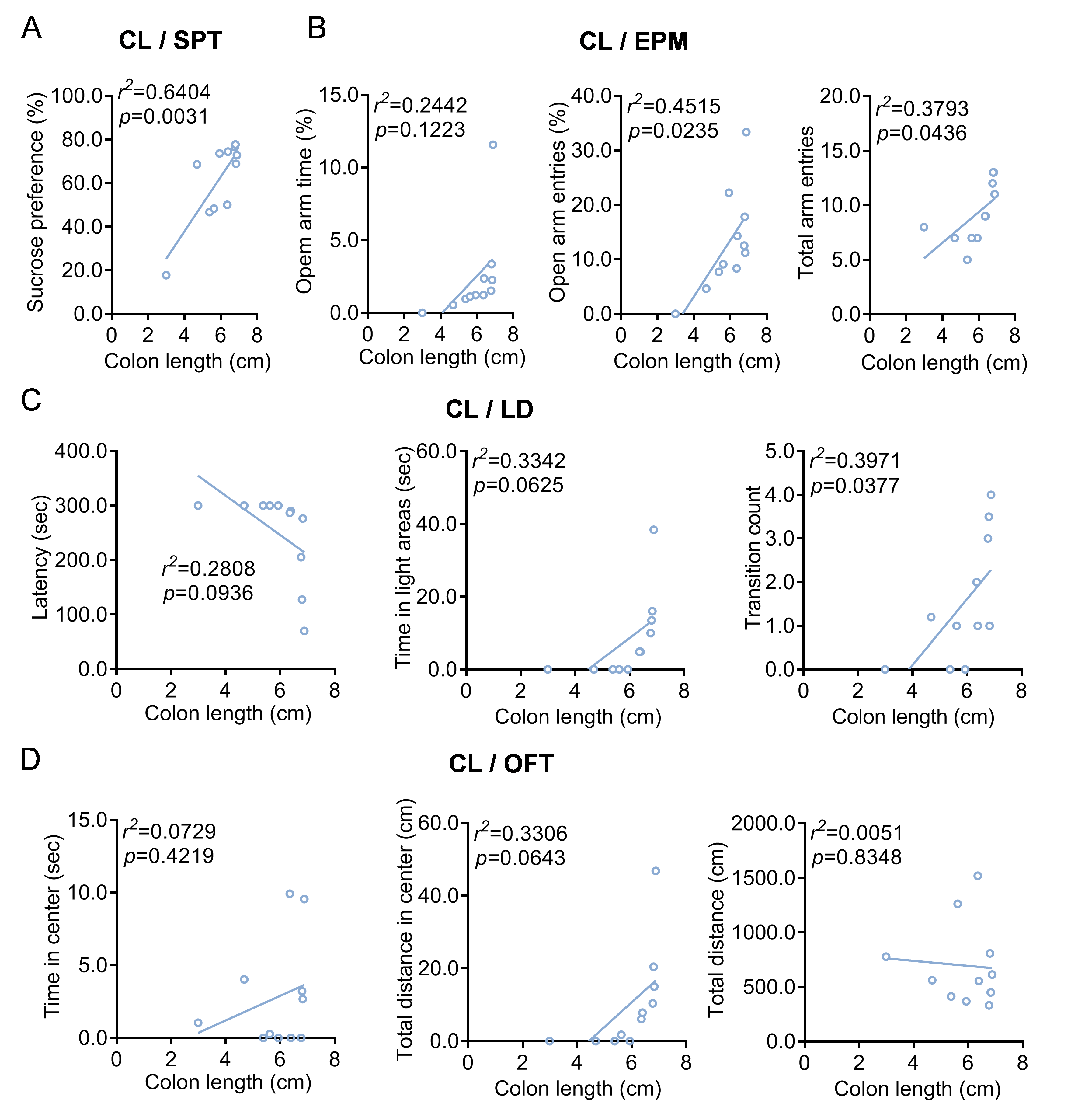

Supplement: Supplementary Figure 4 — The correlation analysis of colonic length with depression/anxiety-related behaviors in the chronic DSS (three days) treated mice. (A) Colonic length (CL)/SPT. (B) Colonic length (CL)/EPM. (C) Colonic length (CL)/LD. (D) Colonic length (CL)/OFT. n=11. [file Image_4.tif]
