# Supplementary material for: The colonic interleukin-19 aggravates the dextran sodium sulfate/stress-induced comorbidities due to colitis and anxiety
Source: Front Immunol. 2023 Mar 2;14:1153344. doi: 10.3389/fimmu.2023.1153344 (PMC10018752; doi:10.3389/fimmu.2023.1153344)
Supplement: Supplementary file 5 [file Table_1.docx]

| **Score** | **Weight loss (%)** | **Stool consistency** | **Occult blood** |
| --- | --- | --- | --- |
| 0 | <0 | Normal | Negative |
| 1 | 1–5 |  | + |
| 2 | 5–10 | Loose | ++ |
| 3 | 10-15 |  | +++ |
| 4 | >15 | Diarrhea | Gross bleeding |

Supplemental table1: Criteria for disease activity index.
