# Supplementary material for: The colonic interleukin-19 aggravates the dextran sodium sulfate/stress-induced comorbidities due to colitis and anxiety
Source: Front Immunol. 2023 Mar 2;14:1153344. doi: 10.3389/fimmu.2023.1153344 (PMC10018752; doi:10.3389/fimmu.2023.1153344)
Supplement: Supplementary file 6 [file Table_2.docx]

Supplemental table2: Primer sequences used for RT-qPCR

| **Gene** | **Forward primer (5'–3')** | **Reverse primer (5'–3')** |
| --- | --- | --- |
| Tubulin | AGCAACATGAATGACCTGGTG | GCTTTCCCTAACCTGCTTGG |
| β-actin | GATCATTGCTCCTCCTGAGC | ACTCCTGCTTGCTGATCCAC |
| IL-19 | TCCTGGCGTTCTACGTGGA | TGACATTGCCGCAGAGTTTTC |
| IL-20Rα | GGCACAAGAGTCTTTGAACCTACTG | GACAGTATGCTCCTGACCCAGG |
| IL-20Rβ | AATGCTCACCGACCAAAAGT | AGGACAGTTGCATTTCGGTT |
| INOS | CAAGAGTTTGACCAGAGGACC | TGGAACCACTCGTACTTGGGA |
| IL-1β | GGCAACTGTTCCTGAACTCAACTG | CCATTGAGGTGGAGAGCTTTCAGC |
| IL-6 | CCACTTCACAAGTCGGAGGCTT | CCAGCTTATCTGTTAGGAGA |
| IL-10 | GCTGGACAACATACTGCTAACC | ATTTCCGATAAGGCTTGGCAA |
| Exon IX | TACCTGGATGCCGCAAACAT | GCTGTGACCCACTCGCTAAT |
| Exon I | CCTGCATCTGTTGGGGAGAC | GCCTTGTCCGTGGACGTTTA |
| Exon II | CTAGCCACCGGGGTGGTGTAA | AGGATGGTCATCACTCTTCTC |
| Exon IV | CAGAGCAGCTGCCTTGATGTT | GCCTTGTCCGTGGACGTTTA |
| Exon VI | CTGGGAGGCTTTGATGAGAC | GCCTTCATGCAACCGAAGTA |
