# Supplementary material for: The colonic interleukin-19 aggravates the dextran sodium sulfate/stress-induced comorbidities due to colitis and anxiety
Source: Front Immunol. 2023 Mar 2;14:1153344. doi: 10.3389/fimmu.2023.1153344 (PMC10018752; doi:10.3389/fimmu.2023.1153344)
Supplement: Supplementary file 7 [file Table_3.docx]

Supplemental table3: GAD-7 scores of IBD patients.

|  | Healthy controls  n=44 | IBD patients  n=32 | P | χ2 | OR | 95% CI |
| --- | --- | --- | --- | --- | --- | --- |
| **Age**  **mean±SEM** | 43.5±1.2700 | 44.8±2.4270 | 0.7200 |  |  |  |
| **Gender,n(%)**  **Female**  **Male** | 23(52.2727)  21(47.7273) | 14(43.7500)  18(56.2500) | 0.4630 | 0.5387 | 0.7101 | 0.2897 to 1.6990 |
| **GAD-7,n(%)**  **mean±SEM** | 7(15.9091)  3.41±0.2374 | 15(46.8750)  5.06±0.6090 | 0.0027 | 8.9890 | 4.7900 | 1.6100 to 12.8100 |
